# The Iranian form of psychometric properties of the Multidimensional Psychological Flexibility Inventory

**DOI:** 10.1186/s41155-022-00236-w

**Published:** 2022-09-29

**Authors:** Zahra Azadfar, Abbas Abdollahi, Indrajit Patra, Ya-Ping Chang, Tawfeeq Alghazali, Saad Ghazi Talib

**Affiliations:** 1 Department of Psychology, Faculty of Educational Sciences and Psychology, Alzahra University, Tehran, Iran; 2 Department of Counseling, Faculty of Education and Psychology, Alzahra University, Tehran, Iran; 3 NIT Durgapur, Durgapur, West Bengal India; 4 Department and Graduate School of Tourism Management, Chinese Culture University, 55, Hwa-Kang Road, Yang Ming Shan, Taipei, Taiwan 11114; 5 Department of Journalism, College of Media, The Islamic University in Najaf, Najaf, Iraq; 6 Law Department, Al-Mustaqbal University College, Babylon, Iraq

**Keywords:** MPFI, Psychological flexibility, Psychological inflexibility, Psychometrics, Iranian

## Abstract

The Multidimensional Psychological Flexibility Inventory (MPFI) is a new 60-item self-report scale developed to assess the specific components of psychological flexibility and inflexibility proposed in the Hexaflex model of Acceptance and Commitment Therapy (ACT). The present study sought to examine the psychometric properties of the Persian version of the MPFI-60 in a community sample of 307 Iranian adults. The original study supported a 12-factor second-order structure consisting of 6 dimensions for psychological flexibility and 6 dimensions for psychological inflexibility. The Persian MPFI-60 demonstrated acceptable semantic and test content, internal structure, correlations with other variables, and internal consistency. It also evidenced in relation to anxiety, stress, depression, and self-compassion. Overall, the results indicate that the Persian MPFI-60 is a psychometrically sound measure in the Iranian context that enables researchers and clinicians to comprehensively assess the components of psychological flexibility and inflexibility within the Hexaflex model.

## Introduction

Psychological flexibility is a transdiagnostic construct that is considered a cornerstone of mental health (Kashdan & Rottenberg, 2010). Psychological flexibility has been conceptualized as the ability to be fully in contact with the present moment, open to inner experiences (e.g., difficult thoughts, feelings, memories, and sensations), and act in accordance with personal goals and values (Hayes et al., 2012). Individuals who have high levels of psychological flexibility are more likely to cope with unpleasant thoughts, emotions, and difficult events in a manner that improves their well-being (Rolffs et al., 2018). Numerous studies have shown that psychological flexibility is associated with well-being, life satisfaction, and lower levels of mental health problems (Kashdan & Rottenberg, 2010; Marshall & Brockman, 2016; Rogge et al., 2019). In addition, psychological flexibility is known to be a protective factor that moderates the relationship between daily life stress, physical functioning, and mental health (Fonseca et al., 2020; Gloster et al., 2017; Pakenham et al., 2020). A growing body of evidence also suggests that psychological flexibility is the key mechanism for change in ACT (Levin et al., 2012), a third-wave behavioral therapy that is an effective treatment for a wide range of mental disorders, including depression, anxiety, chronic pain, eating disorders, substance use, and transdiagnostic conditions (Gloster et al., 2020).

According to the Hexaflex model of ACT (Hayes et al., 1999, 2012), psychological flexibility consists of six interrelated processes: acceptance of difficult inner experiences, diffusion (the ability to observe unwanted inner experiences without getting stuck in them), mindful awareness of the present moment, self-as-context (maintaining a flexible perspective on oneself in the face of difficult experiences), willingness to be in full contact with personal values, and committed action (acting in a value-oriented manner). This model also assumes six dimensions of psychological inflexibility: experiential avoidance (avoiding difficult inner experiences), fusion (getting stuck in undesirable inner experiences rather than observing them), lack of mindful awareness of the present moment, self-as-content (attachment to self-conceptualizations), lack of contact with personal values, and inaction (inability to engage in values-oriented behaviors). Psychological inflexibility is closely related to a variety of mental disorders, including depression (Gilbert et al., 2019), anxiety and somatization (Tavakoli et al., 2019; Venta et al., 2012), eating disorders (Fairburn et al., 2003; Rawal et al., 2010), substance use (Levin et al., 2012), psychosis (Goldstone et al., 2011), and posttraumatic stress disorder (Meyer et al., 2019).

Despite the multidimensional nature of the Hexaflex model, most of the aforementioned studies on the effectiveness of ACT interventions and correlational research on psychological flexibility/inflexibility relied on unidimensional scales, including the Acceptance and Action Questionnaire (AAQ; Hayes et al., 2004), the Acceptance and Action Questionnaire-II (AAQ-II; Bond et al., 2011) and the Brief Experiential Avoidance Questionnaire (BEAQ; Gámez et al., 2014). These scales assess global levels of psychological inflexibility and failed to capture the specific dimensions of the Hexaflex model (Lin et al., 2020; Rolffs et al., 2018; Thomas et al., 2021). Moreover, most items in the above scales are negatively worded to assess psychological inflexibility, and the reversed item scores are used as a measure of psychological flexibility (Rogge et al., 2019). However, Rolffs et al. (2018) assume that psychological flexibility and inflexibility are related, but distinct dimensions, and thus psychological flexibility, cannot be viewed simply as the absence of inflexibility. In addition to the aforementioned limitations, these measures have been criticized for their low construct and discriminant validity. More recently, the AAQ-II and the BEAQ (the most widely used measures of psychological inflexibility) have been shown to capture difficult thoughts and feelings to which individuals respond, rather than measuring individuals’ tendency to react inflexibly to undesirable inner experiences (Kashdan et al., 2020; Landi, Pakenham, Crocetti, et al., 2021; Ong et al., 2020; Tyndall et al., 2019; Wolgast, 2014).

Several multidimensional measures have been developed to assess the specific components of the Hexaflex model of psychological flexibility, including the Multidimensional Experiential Avoidance Questionnaire (MEAQ; Gámez et al., 2011), the Five Facet Mindfulness Questionnaire (FFMQ; Baer et al., 2006), the Self-Compassion Scale (SCS; Neff, 2003), and the Comprehensive Assessment of ACT Processes (CompACT; Francis et al., 2016). Although these multidimensional scales offer the possibility of more accurate measurement of psychological flexibility and inflexibility, they were developed from different conceptual perspectives and focus on some components of the Hexaflex model rather than attempting to comprehensively assess all dimensions of this model (Rolffs et al., 2018; Stabbe et al., 2019; Thomas et al., 2021). The MPFI-60 is the only measure developed in accordance with the Hexaflex model to assess each of the 12 dimensions of psychological flexibility and inflexibility separately (Rolffs et al., 2018).

Rolffs et al. (2018) developed and validated the 60-item MPFI in three studies with a sample of 3040 respondents from the USA. This measure was developed from a pool of 554 potential items, most of which were drawn from 22 widely used measures in the ACT and mindfulness literature. In the first study (*n* = 372), an initial exploratory and confirmatory factor analysis were conducted to determine the factor structure of the MPFI measure. In the second study (*n* = 2150), item response theory (IRT) was applied to a refined pool of 288 items to select the five most effective indicators for each subcomponent of psychological flexibility and inflexibility dimensions. The results of the final exploratory and confirmatory factor analyses revealed a second-order factor structure in which global psychological flexibility and inflexibility are the second-order factors, and the corresponding subcomponents are the first-order factors. In the third study (*n* = 518), convergent validity results indicated that the subscales of the MPFI correlated strongly with existing measures of psychological flexibility and inflexibility. Discriminant validity was also evidenced by the relatively weaker correlations between the subscales of the MPFI and some conceptually distinct constructs (e.g., emotional intelligence, neuroticism, curiosity, need satisfaction, and psychological distress). The 12 subscales of the MPFI showed excellent internal consistencies (*a* = 0.87 to *a* = 0.97) across demographic subgroups, including individuals with different gender, ages, ethnicity, and mental health status (Rolffs et al., 2018).

A subsequent replication study by Seidler et al. (2020) confirmed the second-order factor structure of the MPFI with two general factors (i.e., psychological flexibility and inflexibility) and 12 subscales. In another study, Thomas et al. (2021) examined the factor structure of the MPFI in a large sample of community adults and found support for the higher-order model of MPFI scale. In addition, assessment of the psychometric properties of an Italian version of the MPFI in a sample of 1587 respondents revealed a second-order factor structure for this scale with good internal consistency (*a* = 0.85 to *a* = 0.94) and measurement invariance within different age, gender, and mental health status groups. The concurrent validity of the Italian version of the MPFI has also been demonstrated by strong relationships between psychological flexibility and inflexibility with depression, anxiety, and well-being (Landi, Pakenham, Giovannetti, et al., 2021). Recently, Lin et al. (2020) examined the psychometric properties of the Chinese and Japanese translations of the MPFI in three East Asian countries (i.e., Taiwan, China, and Japan). Confirmatory factor analysis (CFA) supported the higher-order factor structure and measurement invariance of the MPFI across cultural groups and clinical and nonclinical populations. The translated subscales also showed excellent internal consistency (*a* = 0.87 to *a* = 0.94) and convergent correlation patterns with life satisfaction, effective coping, peace of mind, perceived stress, somatic anxiety, and psychological distress.

Since the 60-item MPFI has shown good psychometric properties and strong correlations with indices of mental health and individual functioning in different populations, we aimed to translate it into Persian and investigate its psychometric properties in the Iranian population. As noted by Lin et al. (2020), ACT-based interventions combine Eastern philosophy and Western psychotherapies, with some of their crucial components rooted in Eastern ideologies such as Buddhism and Taoism. However, most of the basic and empirical work examining the components of the Hexaflex model and the benefits of ACT has been conducted in Western cultures. Therefore, the main purpose of the present study was to extend the application of the MPFI beyond Western countries, allowing cross-cultural work on the processes of psychological flexibility and inflexibility in the Hexaflex model. To this end, the CFA was used to examine the factor structure, validity, and reliability of the 60-item MPFI in a sample of Iranian community adults. We hypothesized that the 60-item MPFI would have a second-order factor structure consisting of psychological flexibility (6 items) and inflexibility (6 items) in the Iranian population. The subscales of the MPFI were also examined in relation to stress, anxiety, depression, and self-compassion to ensure the validity of the Persian version of this measure. These constructs were used as convergent and divergent variables in the original psychometric study (Rolffs et al., 2018) and were used as convergent and divergent variables in this study.

## Method

### Participants

The participants in this study were 307 Iranians (226 women and 81 men) aged 18 to 48 years with a mean of 29 and a standard deviation of 9.24. Regarding education, 67 (21.8%) of the participants had a diploma, 142 (46.3%) had a bachelor degrees, 73 (23.8%) had a master degree, and 25 (8.1%) had a Ph.D. In terms of marital status, 202 (65.8%) of them were single, and 105 (34.2%) were married.

### Procedure

The ethics committee of the Alzahra University reviewed and approved the aim and procedures of the study. Surveys were entered into Pors Online forms, and the link was shared on social media for respondents to complete online. The data collection period lasted from August 2021 to February 2022, and it took participants an average of 40 min to complete the online questionnaires.

The Brislin approach (Brislin, 1980) was used to translate the English version of the MPFI-60 into Persian. Two translators who were fluent in both English and Persian translated this measure separately. A translator who was unaware of the translation translated the questionnaire from English into Persian. The questionnaire was then translated into English by a second translator who was unaware of the translation. A comparison was then made between the translated version and the English version, and there was no discrepancy between the two.

### Measures

*The Self-Compassion Scale (SCS)* (Neff, 2003) is a 26-item scale measuring self-compassion and includes six subscales: self-kindness, self-judgment, common humanity, isolation, mindfulness, and overidentification. Response options range from 1 (*almost never*) to 5 (*almost always*). The total self-compassion score is calculated by summing the scores of the six subscales, with higher scores indicating higher levels of self-compassion. An Iranian study conducted by Azizi et al. (2013) showed satisfactory internal consistency for self-compassion with a Cronbach’s alpha of 0.86.

*The Multidimensional Psychological Flexibility Inventory (MPFI)* (Rolffs et al., 2018) is a 60-item measure of psychological flexibility and psychological inflexibility. Psychological flexibility dimension consists of six subscales (acceptance, present moment awareness, self as context, defusion, values, and committed action), with each subscale measured by 5 items. Psychological inflexibility dimension consists of six subscales (experiential avoidance, lack of contact with present moment, self as content, fusion, lack of contact with values, and inaction), with each subscale measured with 5 items. Response options range from 1 (never) to 6 (always). Total scores for psychological flexibility and psychological inflexibility are calculated by summing the scores on the six subscales for each dimension. A higher score in each dimension indicates higher levels of psychological flexibility and psychological inflexibility. Rolffs et al. (2018) showed excellent internal consistency for all subscales of the MPFI-60.

*Depression, Anxiety, Stress Scale-21 (DASS-21)* (Shields et al., 1989) is a 21-item measure of depression, anxiety, and stress. Each of these domains is measured with 7 items. Response options range from 0 (*Did not apply to me at all*) to 3 (*Applied to me very much*), and a higher score on each item indicates greater levels of emotional distress. An Iranian study conducted by Taherifar et al. (2019) showed acceptable internal consistency for depression (*a* = 0.85), anxiety (*a* = 0.83), and stress (*a* = 0.81) (Asghari et al., 2008).

### Data analyses

Semantic analysis refers to the extent to which respondents rated the items’ relevance to the construct and the items’ comprehensibility. The impact score was used to measure item clarity, relevance, and appropriateness using a 5-point response scale ranging from 1 (*not important*) to 5 (*completely important*). The formula for the impact score is the multiplication of frequency and importance. Frequency is the number of respondents who selected a Likert score of 4 and 5, and importance refers to the average Likert score assigned to each item. The item can be considered to have acceptable semantic validity if the impact score is greater than 1.5 (Broder et al., 2007; Hajizadeh & Asghari, 2011). The impact score formula was calculated in the Excel software (version 2016).

Evidence based on test content refers to the experts’ assessment of the appropriateness of the items in assessing of the construct being measured. Content validity index (CVI) and content validity ratio (CVR) were used to measure simplicity, clarity, relevance, and essentiality. The CVI is measured using a 4-point Likert scale ranging from 1 (*not relevant at all*) to 4 (*highly relevant*). The CVI is determined by dividing the total number of experts by the number of experts who selected 3 and 4. If the value of the CVI is above 0.7, this indicates acceptable content validity of the item (Cook & Beckman, 2006). CVR is measured using a 3-point Likert scale ranging from 1 (*not essential*) to 3 (*essential*). The following formula was used to assess test content validity.

$$CVR={n}_e-\left(N/2\right)/\left(N/2\right)$$

In the CVR formula, *n*_*e*_ refers to the experts who chose the number 3 (*essential*), and *N* refers to the total number of experts who rated the content validity of the items. If the CVR value is greater than the Lawshe value (.62) (Lawshe, 1975), it means that the item has acceptable content validity (Cook & Beckman, 2006). The CVI and CVR values were calculated in the Excel software (version 2016).

Evidence based on internal structure was assessed using confirmatory factor analysis in the software AMOS (version 24). Hair Jr et al. (2013) recommended a sample size ratio of cases to number of items of 5:1. The minimum ratio of cases to items was met in this study with 307 cases and 60 items.

In the internal structure assessment phase, factor loading values (according to Kline (2015), acceptable factor loading values are non-negative, less than 1, and greater than 0.5), measurement model fit indices (according to Marsh and Hocevar (1985), CMIN/df between 1 and 5; root mean squared error of approximation (RMSEA) < 0.08; Tucker-Lewis index (TLI), comparative fit index (CFI), and goodness of fit index (GFI) > 0.90, indicating adequate model fit), construct reliability, and convergent validity (the values of average variance extracted (AVE) and construct reliability (CR) were greater than 0.5 and 0.7, respectively, indicating that the measure had acceptable convergent validity and internal consistency (Byrne, 2013)) were measured.

## Results

### Semantic analysis

In this stage, 14 respondents rated the items for relevance, comprehensibility, and appropriateness. Impact scores for the items were then calculated, and the impact score values for all items were greater than 1.5, demonstrating all items maintained in the scale.

### Evidence based on test content

In order to evaluate test content, 12 experts (7 psychologists and 5 consolers) rated the essentiality of items. The values of CVI and CVR were greater than 0.7 and 0.62, respectively (see Table 1), indicating acceptable content validity of the items.

**Table 1:** CVR and CVI for the items of psychological flexibility

No.	Items	CVI			CVR	No.	Items	CVI			CVR
		Simplicity (1–4)	Relevancy (1–4)	Clarity (1–4)	Essential (1–3)			Simplicity (1–4)	Relevancy (1–4)	Clarity (1–4)	Essential (1–3)
**1**	I tried to make peace with my negative thoughts and feelings rather than resisting them	1	1	1	1	31	I tried to distract myself when I felt unpleasant emotions	1	1	1	1
**2**	I experienced myself as separate from my changing thoughts and feelings	1	1	1	0.83	32	When I had a bad memory, I tried to distract myself to make it go away.	1	1	1	1
**3**	I opened myself to all of my feelings, the good and the bad	1	1	1	1	33	When something upsetting came up, I tried very hard to stop thinking about it	1	1	1	1
**4**	I made room to fully experience negative thoughts and emotions, breathing them in rather than pushing them away	1	1	1	1	34	If there was something I didn’t want to think about, I would try many things to get it out of my mind	1	1	1	1
**5**	When I had an upsetting thought or emotion, I tried to give it space rather than ignoring it	0.83	1	1	1	35	When unpleasant memories came to me, I tried to put them out of my mind	1	1	1	1
**6**	I was attentive and aware of my emotions	1	1	1	1	36	I did most things mindlessly without paying much attention	1	1	1	1
**7**	I was in tune with my thoughts and feelings from moment to moment	1	1	1	1	37	I did most things on “automatic” with little awareness of what I was doing	1	1	1	1
**8**	I was in touch with the ebb and flow of my thoughts and feelings	1	1	1	1	38	Most of the time, I was just going through the motions without paying much attention	1	1	1	1
**9**	I paid close attention to what I was thinking and feeling	1	1	1	1	39	I floated through most days without paying much attention	0.92	1	1	1
**10**	I strived to remain mindful and aware of my own thoughts and emotions	1	1	1	1	40	I went through most days on autopilot without paying much attention to what I was thinking or feeling	1	1	1	1
**11**	Even when I felt hurt or upset, I tried to maintain a broader perspective.	0.83	1	0.83	1	41	I thought some of my emotions were bad or inappropriate and I shouldn’t feel them	1	1	1	1
**12**	I carried myself through tough moments by seeing my life from a larger viewpoint	1	1	1	1	42	I criticized myself for having irrational or inappropriate emotions	1	1	1	1
**13**	When I was scared or afraid, I still tried to see the larger picture	0.83	1	0.92	1	43	I believed some of my thoughts are abnormal or bad and I shouldn’t think that way	1	1	1	1
**14**	When something painful happened, I tried to take a balanced view of the situation	1	1	1	1	44	I told myself that I shouldn’t be feeling the way I’m feeling	1	1	1	1
**15**	I tried to keep perspective even when life knocked me down	1	1	0.92	1	45	I told myself I shouldn’t be thinking the way I was thinking	1	1	1	1
**16**	I was able to let negative feelings come and go without getting caught up in them	1	1	1	1	46	Negative thoughts and feelings tended to stick with me for a long time	1	1	1	1
**17**	When I was upset, I was able to let those negative feelings pass through me without clinging to them	1	1	1	1	47	Distressing thoughts tended to spin around in my mind like a broken record	1	1	1	1
**18**	When I was scared or afraid, I was able to gently experience those feelings, allowing them to pass	1	1	1	1	48	It was very easy to get trapped into unwanted thoughts and feelings	1	1	1	1
**19**	In tough situations, I was able to notice my thoughts and feelings without getting overwhelmed by them	1	1	1	1	49	When I had negative thoughts or feelings, it was very hard to see past them	1	1	1	1
**20**	I was able to step back and notice negative thoughts and feelings without reacting to them	1	1	0.92	1	50	When something bad happened, it was hard for me to stop thinking about it	1	1	1	1
**21**	I was very in touch with what is important to me and my life	1	1	1	1	51	When life got hectic, I often lost touch with the things I value	1	1	1	0.83
**22**	I stuck to my deeper priorities in life	1	1	1	1	52	My priorities and values often fell by the wayside in my day-to-day life	1	1	1	1
**23**	I tried to connect with what is truly important to me on a daily basis	1	1	1	1	53	The things that I value the most often fell off my priority list completely	1	1	1	1
**24**	My deeper values consistently gave direction to my life	1	1	1	1	54	When times got tough, it was easy to forget about what I truly value	1	1	1	1
**25**	Even when it meant making tough choices, I still tried to prioritize the things that were important to me	1	1	1	1	55	I didn’t usually have time to focus on the things that are really important to me	1	1	1	1
**26**	Even when times got tough, I was still able to take steps toward what I value in life	1	1	1	1	56	Negative feelings easily stalled out my plans	0.92	1	1	1
**27**	Even when I stumbled in my efforts, I didn’t quit working toward what is important	0.83	1	1	1	57	Negative feelings often trapped me in inaction	1	1	1	1
**28**	Even when life got stressful and hectic, I still worked toward things that were important to me	1	1	1	1	58	Getting upset left me stuck and inactive	1	1	1	1
**29**	I didn’t let setbacks slow me down in taking action toward what I really want in life	1	1	1	1	59	Unpleasant thoughts and feelings easily overwhelmed my efforts to deepen my life	1	1	0.83	1
**30**	I didn’t let my own fears and doubts get in the way of taking action toward my goals	1	1	1	1	60	Negative experiences derailed me from what’s really important	1	1	1	1

### Evidence based on internal structure

In this study, respondents answered the questionnaires via an online link; therefore, there were no missing data in the dataset. A boxplot was used to identify outliers, and the result showed that there were no outliers in the dataset. To assess normality, the values for skewness (−1.08 to 1.21) and kurtosis (1.34 to 2.14) were within the acceptable ranges of ±2 and ±3, respectively (Tabachnick & Fidell, 2012).

**Fig. 1:**
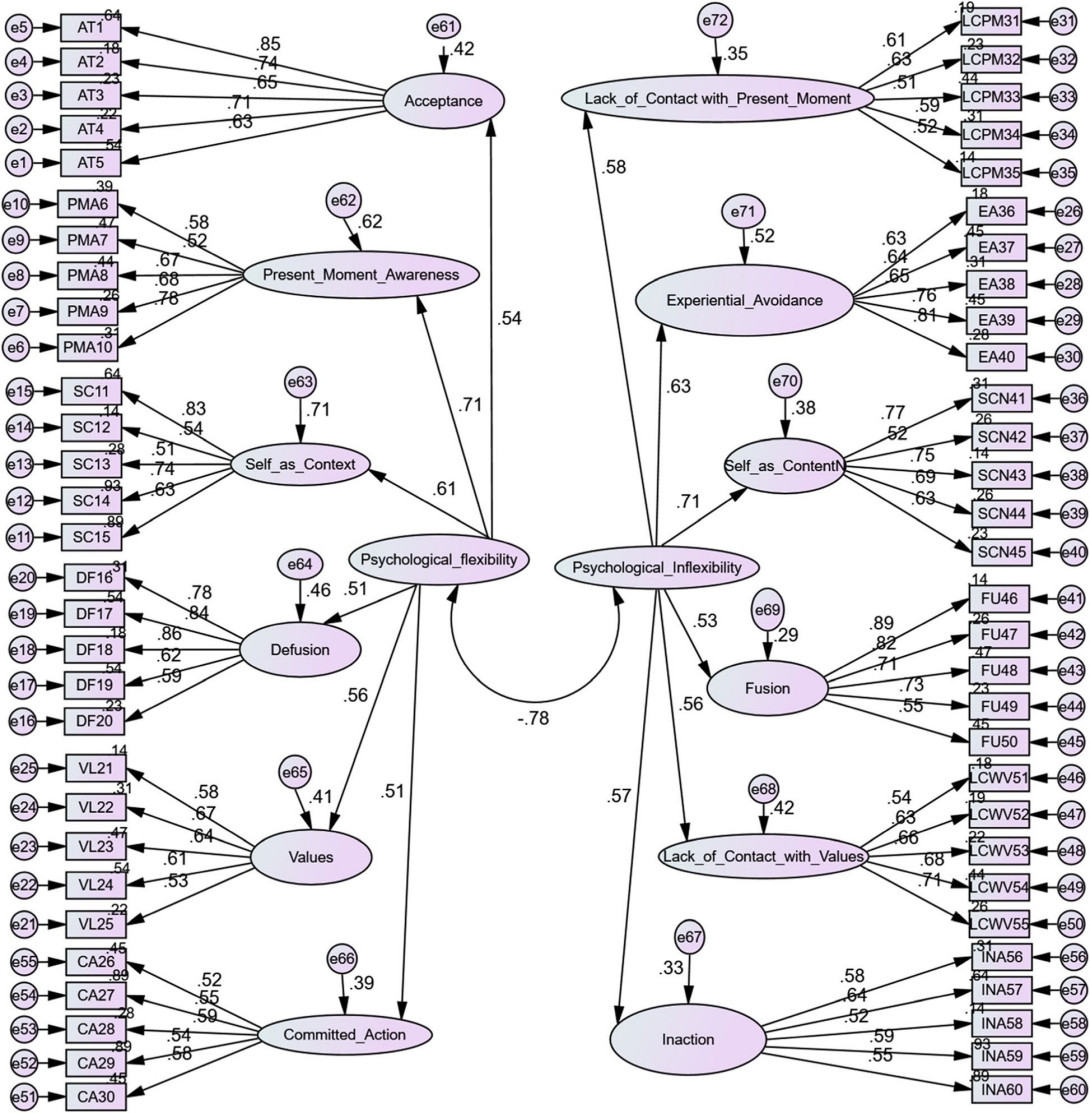
Confirmatory factor analysis with factor loadings for the twelve subscales of the psychological flexibility and psychological inflexibility (*p* < 001). All factor loading values were greater than the cutoff score 0.5. The results of the measurement model fit assessment showed that the fit indices met the cutoff values (CMIN/df = 4.81, *p* < 0.01, CFI = 0.93, RMSEA = 0.06, TLI = 0.92, GFI = 0.92)

Any item having a factor loading of less than 0.5 was to be removed in order to reach the construct’s item quality (Hair et al., 2010). All items were kept in the questionnaire because their factor loadings were greater than 0.5 (see Fig. 1). The means and standard deviations of the items are presented in Table 2. The results of the measurement model fit assessment showed that the fit indices met the cutoff values (*CMIN/df* = 4.81, *p* < 0.01, *CFI* = 0.93, *RMSEA* = 0.06, *TLI* = 0.92, *GFI* = 0.92) and confirmed the twelve subscales of translated MPFI-60. The results of AVE and CR showed that the translated MPFI-60 has acceptable convergent validity and construct reliability (see Table 3).

**Table 2:** Means and standard deviations of the items of psychology flexibility

No.	Items	Mean	Std. deviation	No.	Items	Mean	Std. deviation
1	I tried to make peace with my negative thoughts and feelings rather than resisting them	3.47	1.12	31	I tried to distract myself when I felt unpleasant emotions	3.59	1.27
2	I experienced myself as separate from my changing thoughts and feelings	2.47	1.41	32	When I had a bad memory, I tried to distract myself to make it go away	3.61	1.32
3	I opened myself to all of my feelings, the good and the bad	4.13	1.23	33	When something upsetting came up, I tried very hard to stop thinking about it	3.43	1.38
4	I made room to fully experience negative thoughts and emotions, breathing them in rather than pushing them away	3.48	1.31	34	If there was something I didn’t want to think about, I would try many things to get it out of my mind	3.83	1.25
5	When I had an upsetting thought or emotion, I tried to give it space rather than ignoring it	3.51	1.34	35	When unpleasant memories came to me, I tried to put them out of my mind	3.67	1.31
6	I was attentive and aware of my emotions	4.52	1.07	36	I did most things mindlessly without paying much attention	2.38	1.06
7	I was in tune with my thoughts and feelings from moment to moment	4.47	1.04	37	I did most things on “automatic” with little awareness of what I was doing	2.79	1.17
8	I was in touch with the ebb and flow of my thoughts and feelings	4.46	1.01	38	Most of the time, I was just going through the motions without paying much attention	2.39	1.03
9	I paid close attention to what I was thinking and feeling	4.68	1.05	39	I floated through most days without paying much attention	2.91	1.33
10	I strived to remain mindful and aware of my own thoughts and emotions	4.34	1.11	40	I went through most days on autopilot without paying much attention to what I was thinking or feeling	2.48	1.08
11	Even when I felt hurt or upset, I tried to maintain a broader perspective	3.88	1.23	41	I thought some of my emotions were bad or inappropriate and I shouldn’t feel them	3.23	1.35
12	I carried myself through tough moments by seeing my life from a larger viewpoint	4.14	1.25	42	I criticized myself for having irrational or inappropriate emotions	2.88	1.48
13	When I was scared or afraid, I still tried to see the larger picture	3.69	1.32	43	I believed some of my thoughts are abnormal or bad and I shouldn’t think that way	3.23	1.46
14	When something painful happened, I tried to take a balanced view of the situation	3.86	1.29	44	I told myself that I shouldn’t be feeling the way I’m feeling	3.14	1.48
15	I tried to keep perspective even when life knocked me down	3.91	1.28	45	I told myself I shouldn’t be thinking the way I was thinking	3.15	1.49
16	I was able to let negative feelings come and go without getting caught up in them	3.07	1.19	46	Negative thoughts and feelings tended to stick with me for a long time	3.21	1.46
17	When I was upset, I was able to let those negative feelings pass through me without clinging to them	2.96	1.24	47	Distressing thoughts tended to spin around in my mind like a broken record	3.01	1.50
18	When I was scared or afraid, I was able to gently experience those feelings, allowing them to pass	3.28	1.19	48	It was very easy to get trapped into unwanted thoughts and feelings	3.05	1.41
19	In tough situations, I was able to notice my thoughts and feelings without getting overwhelmed by them	3.22	1.26	49	When I had negative thoughts or feelings, it was very hard to see past them	3.31	1.38
20	I was able to step back and notice negative thoughts and feelings without reacting to them	3.24	1.19	50	When something bad happened, it was hard for me to stop thinking about it	3.76	1.32
21	I was very in touch with what is important to me and my life	4.65	1.03	51	When life got hectic, I often lost touch with the things I value	2.57	1.18
22	I stuck to my deeper priorities in life	4.79	1.06	52	My priorities and values often fell by the wayside in my day-to-day life	2.37	1.15
23	I tried to connect with what is truly important to me on a daily basis	4.58	1.07	53	The things that I value the most often fell off my priority list completely	2.30	1.13
24	My deeper values consistently gave direction to my life	4.63	1.10	54	When times got tough, it was easy to forget about what I truly value	2.21	1.12
25	Even when it meant making tough choices, I still tried to prioritize the things that were important to me	4.48	1.09	55	I didn’t usually have time to focus on the things that are really important to me	2.40	1.08
26	Even when times got tough, I was still able to take steps toward what I value in life	4.45	1.12	56	Negative feelings easily stalled out my plans	3.02	1.31
27	Even when I stumbled in my efforts, I didn’t quit working toward what is important	4.33	1.30	57	Negative feelings often trapped me in inaction	2.89	1.35
28	Even when life got stressful and hectic, I still worked toward things that were important to me	4.29	1.17	58	Getting upset left me stuck and inactive	3.16	1.34
29	I didn’t let setbacks slow me down in taking action toward what I really want in life	4.24	1.15	59	Unpleasant thoughts and feelings easily overwhelmed my efforts to deepen my life	2.89	1.32
30	I didn’t let my own fears and doubts get in the way of taking action toward my goals	4.15	1.20	60	Negative experiences derailed me from what’s really important	2.81	1.27

**Table 3:** AVE and CR for twelve subscales of psychological flexibility

Variable	AVE	CR
Psychological flexibility	0.62	0.76
Acceptance (items 1 to 5)	0.64	0.72
Present moment awareness (items 6 to 10)	0.64	0.78
Self as context (items 11 to 15)	0.71	0.81
Defusion (items 16 to 20)	0.68	0.79
Values (items 21 to 25)	0.53	0.71
Committed action (items 26 to 30)	0.51	0.73
Psychological inflexibility	0.65	0.74
Experiential avoidance (items 31 to 35)	0.56	0.73
Lack of contact with present moment (items 36 to 40)	0.57	0.73
Self as content (items 41 to 45)	0.61	0.71
Fusion (items 46 to 50)	0.69	0.71
Lack of contact with values (items 51 to 55)	0.71	0.75
Inaction (items 56 to 60)	0.74	0.81

### Evidence based on relations to other variables

Pearson correlation analysis between the Persian translated version of MPFI-60 with depression, anxiety, and stress was assessed in this step. The results showed that the six subscales of psychological flexibility were negatively associated with depression, anxiety, and stress and positively associated with self-compassion. The results also showed that the six subscales of psychological inflexibility were positively associated with depression, anxiety, and stress and negatively associated with self-compassion (see Table 4).

**Table 4:** Correlations between the studied variables

Variables	1	2	3	4	5	6	7	8	9	10	11	12	13	14	15	16	17	18
1) Psychological flexibility	1																	
2) Acceptance	0.54	1																
3) Present moment awareness	0.69	0.56	1															
4) Self-as-context	0.60	0.48	0.48	1														
5) Defusion	0.52	0.57	0.51	0.47	1													
6) Values	0.59	0.42	0.44	0.38	0.38	1												
7 7) Committed action	0.54	0.41	0.39	0.42	0.41	0.54	1											
8) Psychological inflexibility	−0.79	−0.57	−0.49	−0.51	−0.43	−0.41	−0.43	1										
9) Experiential avoidance	−0.52	−0.51	−0.38	−0.48	−0.39	−0.38	−0.52	0.61	1									
10) Lack of contact with present moment	−0.56	−0.62	−0.47	−0.57	−0.38	−0.41	−0.42	0.57	0.55	1								
11) Self as content	−0.61	−0.63	−0.41	−0.52	−0.36	−0.52	−0.39	0.72	0.49	0.52	1							
12) Fusion	−0.63	−0.57	−0.43	−0.53	−0.35	−0.55	−0.49	0.53	0.53	0.58	0.44	1						
13) Lack of contact with values	−0.51	−0.59	−0.45	−0.48	−0.44	−0.49	−0.47	0.56	0.57	0.59	0.49	0.59	1					
14) Inaction	−0.51	−0.45	−0.47	−0.61	−0.43	−0.37	−0.37	0.57	0.58	0.63	0.51	0.58	0.44	1				
15) Depression	−0.49	−0.42	−0.51	−0.62	−0.45	−0.55	−0.51	0.51	0.61	0.68	0.52	0.49	0.49	0.55	1			
16) Anxiety	−0.46	−0.41	−0.55	−0.67	−0.39	−0.63	−0.47	0.52	0.49	0.57	0.49	0.44	0.58	0.63	0.58	1		
17) Stress	−0.51	−0.44	−0.49	−0.53	−0.43	−0.46	−0.42	0.44	0.52	0.49	0.56	0.41	0.62	0.58	0.51	0.54	1	
18) Self-compassion	0.42	0.47	0.49	0.52	0.42	0.43	0.58	−0.43	−0.52	−0.39	−0.58	−0.47	−0.51	−0.47	−0.51	−0.40	−0.39	1
All paths are significant at the level of 0.001																		

## Discussion

The present study aimed to extend research on multidimensional psychological flexibility and inflexibility to Eastern populations by translating the MPFI-60 into Persian and assessing its psychometric properties among an Iranian community sample of adults. The results of the semantic validity assessment using the impact score index confirmed the relevance, comprehensibly, and appropriateness of the translated MPFI-60 indicators. The results of quantitative content validity also showed acceptable content validity of the MPFI-60 items. Confirmatory factor analysis, consistent with previous psychometric work (Landi, Pakenham, Giovannetti, et al., 2021; Lin et al., 2020; Rolffs et al., 2018; Seidler et al., 2020; Thomas et al., 2021) and the Hexaflex model (Hayes et al., 1999, 2012), yielded a second-order factor structure consisting of six first-order factors for psychological flexibility (i.e., acceptance, present moment awareness, self as context, defusion, values, and committed action) and six first-order factors for psychological inflexibility (i.e., experiential avoidance, lack of contact with present moment, self as content, fusion, lack of contact with values, and inaction). The factor loading values of all indicators were above 0.5 (ranging from 0.51 to 0.89), so they all remained on the scale. The values of CR (within the range of 0.71 to 0.81) and AVE (within the range of 0.51 to 0.74) supported the acceptable construct reliability and convergent validity of the MPFI-60, respectively.

The results of the correlation analysis support the Hexaflex model (Hayes et al., 1999, 2012) and the findings of previous research (Landi, Pakenham, Giovannetti, et al., 2021; Lin et al., 2020; Rolffs et al., 2018; Stabbe et al., 2019) suggesting that global psychological flexibility and psychological inflexibility and their subscales are related but distinct constructs that may change independently. The intercorrelations of the six flexibility subprocesses in Table 4 show moderate correlations (ranging from 0.38 to 0.57) with an average common variance of 0.21, similar to those reported by Rolffs et al. (2018). Although moderately correlated, each of the dimensions of psychological flexibility contains meaningful unique variance, and improvements in one dimension of psychological flexibility (e.g., acceptance) are not necessarily accompanied by improvements in other dimensions. Similarly, the dimensions of psychological inflexibility correlate sensibly from 0.44 to 0.63 with each other (average common variance of 0.29), similar to the range (0.31 to 0.78) reported in the Rolffs's et al. (2018) study. Accordingly, an inflexible client may be high on experiential avoidance but lower on other dimensions of psychological inflexibility. Moreover, the dimensions of flexibility are moderately correlated (−0.35 to −0.63) but distinct from their inflexibility counterparts. This implies that psychological flexibility and inflexibility are not simply two opposite ends of a single dimension but two distinct processes that should be considered independently.

Convergent validity of the translated MPFI-60 was evidenced by significant positive relationships between global psychological inflexibility and its subscales with indicators of emotional distress (i.e., anxiety, stress, and depressive symptoms) and by opposite patterns of relationships for global psychological flexibility and its subscales. As hypothesized, higher levels of acceptance, present moment awareness, self as context, defusion, values, and committed action were negatively associated with anxiety, stress, and depressive symptoms. Higher scores on experiential avoidance, lack of contact with present moment, self as content, fusion, lack of contact with values, and inaction were positively associated with higher levels of anxiety, stress, and depressive symptoms. These results are in line with findings from previous research on the MPFI-60 (Landi, Pakenham, Giovannetti, et al., 2021; Lin et al., 2020; Rogge et al., 2019; Rolffs et al., 2018; Stabbe et al., 2019; Thomas et al., 2021). Moreover, scores on the global psychological flexibility and its subscale were positively associated with higher scores on self-compassion. In contrast, psychological inflexibility and its subscales were negatively related to self-compassion. This finding is consistent with previous studies (Farr et al., 2021; Marshall & Brockman, 2016; Mendes et al., 2022; Pyszkowska & Rönnlund, 2021; Rolffs et al., 2018) indicating that processes of psychological flexibility and inflexibility are related to self-compassion attitudes.

### Implications

This study provides initial support for the validity and reliability of the Persian version of the MPFI-60 for a comprehensive assessment of specific dimensions of psychological flexibility and inflexibility within the Hexaflex model. The MPFI-60 would offer researchers a method of assessing potential mechanisms of change to determine which components of the Hexaflex model are more strongly related to mental health and well-being and which are more influenced by the ACT interventions. In addition, the flexibility/inflexibility profiles provided by MPFI-60 allow ACT therapists to get a more nuanced picture of each client’s current level of functioning and unique challenges to determine potential treatment goals.

### Limitations and future research

Several limitations should be considered when interpreting the results of the present study. First, the data for this study were collected via an online survey and self-report questionnaires. Second, the sample was recruited using the convenience sampling method and contained predominantly female participants, which limits the generalizability of the findings. Future research should examine the psychometric properties of the MPFI-60 in a more diverse population. Third, this study was conducted on a general sample of adults. Future research could validate the Persian version of the MPFI-60 on a clinical sample currently receiving psychotherapies for mental health problems. Forth, this study was cross-sectional, so it is suggested that future longitudinal studies examine the test-retest reliability of the Persian version of the MPFI-60 and the sensitivity of its subscales to detect changes in flexibility and inflexibility over time.

## Conclusion

The current study was a critical step in supporting the validity and reliability of the most comprehensive measure for assessing specific components of flexibility and inflexibility in the Iranian population. The findings show that the MPFI-60 is a reliable and valid measure for assessing the specific dimensions of psychological flexibility and inflexibility in the Iranian population. It should be noted that validity is not a property of the tool itself but rather of the interpretation or specific purpose of the assessment tool with particular settings. Therefore, this measure may open new lines of research on the relationships between the subcomponents of the Hexaflex model and psychological outcomes, as well as on the effectiveness of ACT interventions in Persian-speaking populations.

## Data Availability

The datasets generated during and/or analyzed during the current study are available in the Figshare repository, (10.6084/m9.figshare.20006429).

## Abbreviations

MPFI: The Multidimensional Psychological Flexibility Inventory
ACT: Acceptance and commitment therapy
AAQ: Acceptance and Action Questionnaire
AAQ-II: Acceptance and Action Questionnaire-II
BEAQ: Brief Experiential Avoidance Questionnaire
MEAQ: Multidimensional Experiential Avoidance Questionnaire
FFMQ: Five Facet Mindfulness Questionnaire
SCS: Self-Compassion Scale
CompACT: Comprehensive Assessment of ACT Processes
IRT: Item response theory
CFA: Confirmatory factor analysis
DASS-21: Depression, Anxiety, Stress Scale-21
CVI: Content validity index
CVR: Content validity ratio
RMSEA: Root mean squared error of approximation
TLI: Tucker-Lewis index
CFI: Comparative fit index
GFI: Goodness of fit index
AVE: Average variance extracted
CR: Construct reliability
